# The characteristics of laboratory tests at admission and the risk factors for adverse clinical outcomes of severe and critical COVID-19 patients

**DOI:** 10.1186/s12879-021-06057-z

**Published:** 2021-04-20

**Authors:** Liulin Wang, Xiaobin Cheng, Qiufen Dong, Chenliang Zhou, Yeming Wang, Bin Song, Weinan Li, Min Wang, Rui Qin, Qi Long, Juan Liu, Jing Li, Dan Li, Gang Li, Yuanming Ba

**Affiliations:** 1 Department of Critical Care Medicine, Hubei Provincial Hospital of Tranditional Chinese Medicine, Wuhan, China; 2 Hubei Provincial Academy of Tranditional Chinese Medicine, Wuhan, China; 3 Department of Critical Care Medicine, Renmin Hospital of Wuhan University, Wuhan, China; 4 Department of Critical Care Medicine, Hubei Provincial Hospital of Integrated Chinese & Western Medicine, Wuhan, China; 5 Department of Critical Care Medicine, Jin Yin-tan Hospital, Wuhan, China; 6 Nephrology Department, Hubei Provincial Hospital of Tranditional Chinese Medicine, Wuhan, China

**Keywords:** COVID-19, Laboratory tests, Cox proportional hazards model, Risk factors

## Abstract

**Background:**

The current coronavirus disease 2019 (COVID-19) is a public health emergency. In this study, we aimed to evaluate the risk factors for mortality in severe and critical COVID-19 patients.

**Methods:**

We performed a retrospective study of patients diagnosed with severe and critical COVID-19 from four hospitals in Wuhan, China, by evaluating the clinical characteristics and laboratory results, and using Cox proportional hazards model to assess the risk factors involved in disease progression.

**Results:**

In total, 446 patients with COVID-19 were enrolled. The study indicated a high mortality rate (20.2%) in severe and critical COVID-19 patients. At the time of admission, all patients required oxygen therapy, and 52 (12%) required invasive mechanical ventilation, of which 50 (96%) died. The univariate Cox proportional hazards model showed a white blood cell count of more than 10 × 10^9^/L (HR 3.993,95%CI 2.469 to 6.459) that correlated with an increased mortality rate. The multivariable Cox proportional hazards model demonstrated that older age (HR 1.066, 95% CI 1.043 to 1.089) and higher white blood cell count (HR 1.135, 95% CI 1.080 to 1.192) were independent risk factors for determining COVID-19 associated mortality.

**Conclusions:**

COVID-19 is associated with a significant risk of morbidity and mortality in the population. Older age and higher white blood cell count were found to be independent risk factors for mortality.

## Background

In December 2019, a cluster of patients with pneumonia of unknown etiology was identified in Wuhan City, Hubei Province, China. Isolation of bronchoalveolar-lavage samples from patients with pneumonia in human airway epithelial cells and subsequent whole-genome sequencing revealed the presence of a novel beta-coronavirus called severe acute respiratory syndrome coronavirus 2 (SARS-CoV-2) [1], and the viral disease was officially named coronavirus disease 2019 (COVID-19) on February 12, 2020 [2]. The virus spread internationally at a rapid rate within a month after its first identification; and the virus is mostly transmitted through respiratory droplets and close contact with infected people [3]. Severe acute respiratory syndrome (caused by SARS-CoV) [4–6], which occurred in 2003, and Middle East respiratory syndrome coronavirus (MERS -CoV), which occurred in 2013, belongs to the same beta-coronavirus genus of coronaviruses as SARS-CoV-2 [7, 8]. They are highly pathogenic and the infection manifests as a severe acute respiratory disease [9]. According to bioinformatics prediction methods and in vitro experiments, it was found that the virus may attach to host cells through the human angiotensin-converting enzyme 2 (ACE2) receptor found primarily in airway epithelial cells [10, 11].

To date, more than 2 million deaths of the pandemic and huge economic and social upheaval internationally [12]. There is no effective therapy for COVID-19,vaccine is the best defense against COVID-19. In order to explore the onset of clinical characteristics of COVID-19 and assess the prognostic risk factors for patients, we conducted laboratory tests for 446 patients, evaluated the short-term results, and tried to identify the possible clinical consequences and final outcome of the patients.

## Methods

### Study design and participants

All methods in this study were performed in accordance with the relevant guidelines and regulations. The study was approved by the Institutional Review Board (IRB) at the Hubei Provincial Hospital of Traditional Chinese Medicine (HBZY2020-C14–01) in Wuhan, P. R. China. Oral consent was obtained from patients or patients’ relatives. All cases came from Hubei Provincial Hospital of Traditional Chinese Medicine, Renmin Hospital of Wuhan University, Hubei Provincial Hospital of Integrated Chinese & Western Medicine, and JinYin-tan Hospital (Wuhan, China). The first patient was admitted on January 15, 2020 and the follow-up deadline for the last admitted patients was March 1, 2020. Also included in our study were 31 severe and critical patients in a previous publication [13].

All patients who were enrolled in this study were diagnosed with COVID-19 according to the guidance provided by the Chinese National Health Commission. The cases were classified as either severe or critical. The clinical classification was based on the fifth trial version of the Diagnosis and Treatment Scheme for Pneumonitis with COVID-19 infection, released by China’s National Health Commission. Based on this scheme, one of the following conditions must be met for a patient to be classified as severe: ① shortness of breath, respiratory rate ≥ 30 beats/min, ② resting state, oxygen saturation ≤ 93%, ③ arterial blood oxygen partial pressure (PaO_2_)/oxygen concentration (FiO2) ≤ 300 mmHg, or ④ pulmonary imaging of blood showing significant progression of lesions > 50%. Critical patients must show one of the following: ① respiratory failure, requiring mechanical ventilation, ② shock, or ③combined failure of other organs requiring ICU monitoring and treatment (Table 1).

**Table 1:** Criteria for diagnosis of severe and critical illness

Severe	Critical
① Shortness of breath, respiratory rate ≥ 30 beats / min	① Respiratory failure, requiring mechanical ventilation
② Resting state, oxygen saturation ≤ 93%	② Shock
③Arterial blood oxygen partial pressure (PaO2)/oxygen concentration (FiO2) ≤ 300 mmHg	③ Combined failure of other organs requiring ICU monitoring and treatment
④Pulmonary imaging of blood showing significant progression of lesions > 50%	

Severe and critical illness meet any of the above

Outcome indicators included discharge after treatment (survival) and death. The patient was discharged based on meeting the following four criteria: ① no fever for at least three days, ② significant improvement in both lungs on a chest computed tomography (CT) scan, ③ clinical relief from respiratory symptoms, and ④ two sequential SARS-CoV-2 RNA negative throat swab samples obtained at least 24 h apart (Table 2).

**Table 2:** Discharge diagnosis criteria

① No fever for at least three days	
② Significant improvement in both lungs on a chest computed tomography (CT) scan	
③ Clinical relief from respiratory symptoms	
④ Two sequential SARS-CoV-2 RNA negative throat swab samples obtained at least 24 h apart	

The above four conditions must be met at the same time

### Data collection

All data were collected by specialists with extensive clinical experience. Electronic medical records were used to collect data on general vital signs, clinical symptoms, underlying diseases (diabetes, hypertension, and coronary heart disease), laboratory tests, and treatment outcomes of patients at the time of admission. The collected data were collated and reviewed by a team of professionally trained doctors. The collated experimental data included blood analysis (white blood cell count, lymphocyte count, and lymphocyte percentage), liver function (levels of glutamate transferase and aspartate transferase), renal function (levels of blood urea nitrogen, creatinine, and uric acid), and the levels of glucose, triglycerides, cholesterol, high-sensitivity cardiac troponin I, interleukin-6 (IL-6), procalcitonin, and C-reactive protein (CRP).

### Statistical analysis

Statistical analysis was performed using R version 4.0.2. Categorical variables were described by frequency and percentage, and continuous variables were described by median and interquartile range (IQR) values. Continuous variables were compared using independent group tests when the data were normally distributed; otherwise, the Mann-Whitney test was used.

The Cox proportional hazards model was used to analyze baseline variables related to mortality. For each continuous variable (e.g. glucose, AST, age and white blood cell counts), martingale residuals were utilized to assess each variable’s functional form. It turned out that the log transformed format is more appropriate to glucose and AST. Thus, the log transformed glucose and AST were included in the analyses. Variables that were found to be statistically significant (*p* < 0.05) in univariate analysis were included in the multivariable analysis. The Kaplan-Meier method was used to analyze the survival rate, and the log-rank test was used to compare the survival rates. We tested in the model interactions that were significant on a stratified analysis hazards ratio (HR) presented with 95% confidence intervals (CI). Testing for the proportional hazards assumption was conducted by checking the scaled Schoenfeld residuals using Survival package 3.2–3 in R version 4.0.2. The results showed that all variables satisfied the assumption. All statistical tests were mutually exclusive and statistical significance was set at *p* < 0.05.

## Results

### General characteristics

The median age of the 446 patients included in the study was 55 years (IQR 42–66); of which, 213 (47.76%) were male, 90 (20.2%) died during hospitalization, and 356 (79.8%) were discharged. The median length of hospital stay was 10 days (IQR 8–14) (Table 3). A total of 104 (23.3%) patients that were diagnosed with one or more chronic diseases: 84 (18.83%) were diabetic, 104 (23.3%) were hypertensive, and 35 (7.8%) exhibited coronary heart disease. On admission, hypoxemia was a common symptom observed, so all patients received oxygen therapy, 394 (88%) patients required high-flow nasal cannula oxygen therapy or noninvasive mechanical ventilation, while 52 (17%) patients required invasive mechanical ventilation, of which 50 (96%) died. (Table 3).

**Table 3:** Demographic, clinical, and laboratory of patients on admission

	Total(*N* = 446)	Alive (*N* = 356)	Dead(*N* = 90)
Sex			
Male	213/446 (48%)	161/356 (45%)	52/90 (58%)
female	233/446 (52%)	195/356 (55%)	38/90 (42%)
Age (years)	55/446 (42–66)	50 (40–61)	69 (62–78)
>60	179/446 (40%)	104/356 (29%)	75/90 (83%)
Oxygen therapy			
HFNC,NMV	394/446 (88%)	354/356 (99%)	40/90 (44%)
IMV	52/446 (12%)	2/356 (1%)	50/90 (56%)
Diabetes	84/446 (19%)	57/356 (16%)	27/90 (30%)
Hypertension	104/446 (23%)	73/356 (21%)	31/90 (34%)
Coronary heart disease	35/446 (7.8%)	22/356 (6.18%)	13/90 (14.44%)
Hospital length of stay (days)	10 (8–14)	10 (8–13)	9 (5–12)
White blood cell count (×10^9^per L)	5.50 (4.10–7.62)	5.18 (3.95–7.05)	7.56 (5.34–10.95)
<4	105/446 (23%)	92/356 (26%)	13/90 (14%)
4–10	289/446 (65%)	239/356 (67%)	50/90 (56%)
>10	52/446 (12%)	25/356 (7%)	27/90 (30%)
Lymphocytes (×10^9^per L)	1.01 (0.68–1.41)	1.12 (0.77–1.58)	0.66 (0.44–0.89)
<1	218/446 (49%)	145/356 (41%)	73/90 (81%)
≥ 1	228/446 (51%)	211/356 (59%)	17/90 (19%)
Lymphocyte percentage (%)	16.7 (3.95–27.60)	20.75 (6.83–29.80)	5.10 (0.31–11.50)
<20	253/446 (57%)	173/356 (49%)	80/90 (89%)
≥ 20	193/446 (43%)	183/356 (51%)	10/90 (11%)
ALT (U/L)	25 (16–42)	24 (16–40)	26 (16–50)
>40	121/446 (27%)	84/356 (24%)	37/90 (41%)
AST (U/L)	27 (19–40)	25 (18–37)	26 (16–50)
>40	111/446 (25%)	65/356 (18%)	46/90 (51%)
BUN (mmol/L)	4.5 (3.5–6.0)	4.2 (3.3–5.5)	6.97 (4.8–10.1)
>9.5	32/446 (7%)	7/356 (2%)	25/90 (28%)
Creatinine, (μmol/L)	63.7 (52.1–80.0)	4.2 (3.3–5.5)	73.1 (56.1–99.4)
>104	30/446 (7%)	12/356 (3%)	18/90 (20%)
UA (μmol/L)	245 (187–305)	247 (194–315	242.00 (153–293)
Glucose (mmol/L)	5.8 (4.9–7.3)	5.6 (4.9–6.7)	7.2 (5.9–9.7)
>6.1	200/446 (45%)	136/356 (38%)	64/90 (71%)
3.9–6.1	235/446 (53%)	210/356 (59%)	25/90 (28%)
<3.9	11/446 (2%)	10/356 (3%)	1/90 (1%)
Triglyceride (mmol/L)	1.3 (1.0–1.7)	1.3 (1.0–1.7)	1.3 (1.1–1.6)
Cholesterol (mmol/L)	3.9 (3.4–4.4)	3.9 (3.4–4.5)	3.8 (3.2–4.3)
High-sensitivity cardiac troponin I (ng/mL)	0.006 (0.02–0.18)	0.006 (0.002–0.008)	0.02 (0.007–0.038)
IL-6 (pg/mL)	9.5 (6.1–22.0)	7.8 (5.7–15.5)	12.1 (8.9–25.4)
Procalcitonin (ng/mL)	0.05 (0.05–0.13)	0.05 (0.04–0.09)	0.14 (0.06–0.34)
C-reactive protein (mg/L)	26.8 (5.0–78.5)	18.2 (5.0–49.0)	84.3 (28.8–155.5)
>10	256/380 (67%)	178/300 (59%)	78/80 (98%)

### Laboratory test results

At the time of admission, 105 (23%) patients presented a leukocyte count below the normal range (white blood cell count less than 4 × 10^9^/L), 52 (12%) with a leukocyte count above the normal range (white blood cell count more than 10 × 10^9^/L), and about 50% had a decreased lymphocyte count (lymphocyte count less than 1.0 × 10^9^/L). A total of 121 (27%) patients exhibited varying degrees of liver dysfunction, with alanine aminotransferase (ALT) or aspartate aminotransferase (AST) levels above the normal range. Renal dysfunction occurred in 32 (7%) patients, who showed elevated blood urea nitrogen or serum creatinine levels. There were 211 (47%) patients with abnormal blood glucose levels, and among them, 200 (45%) showed glucose levels above the normal range (glucose concentration more than 6.1 mmol/L), while 11 (2%) patients showed levels below the normal range (glucose concentration less than 3.9 mmol/L). Levels of CRP were increased in 67% of the patients (CRP more than 10 mg/L) (Table 3). The results of other laboratory tests are shown in Table 3.

### Factors affecting the mortality rate

**Fig. 1:**
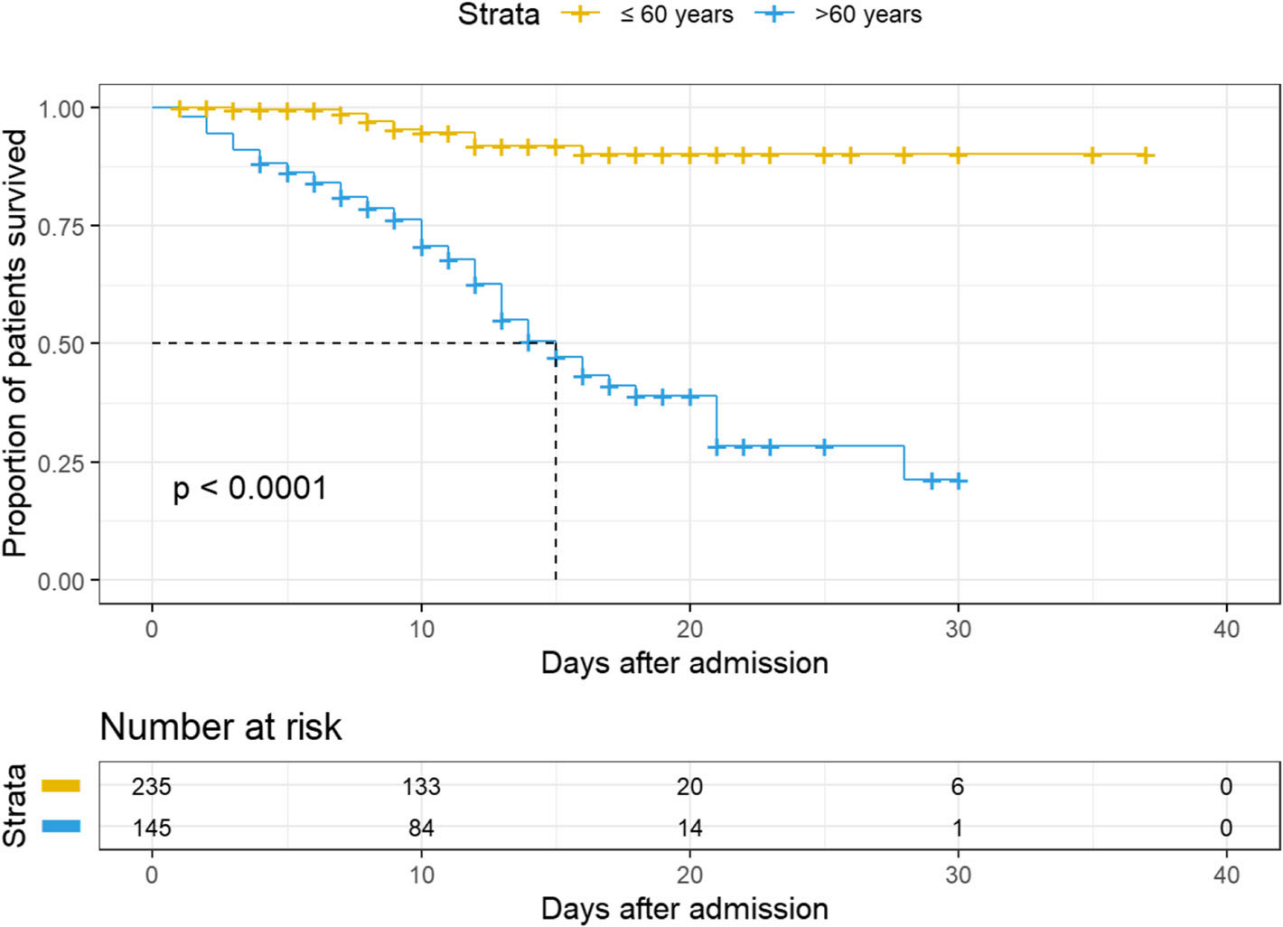
Kaplan-Meier Survival Curves by Age Group

**Fig. 2:**
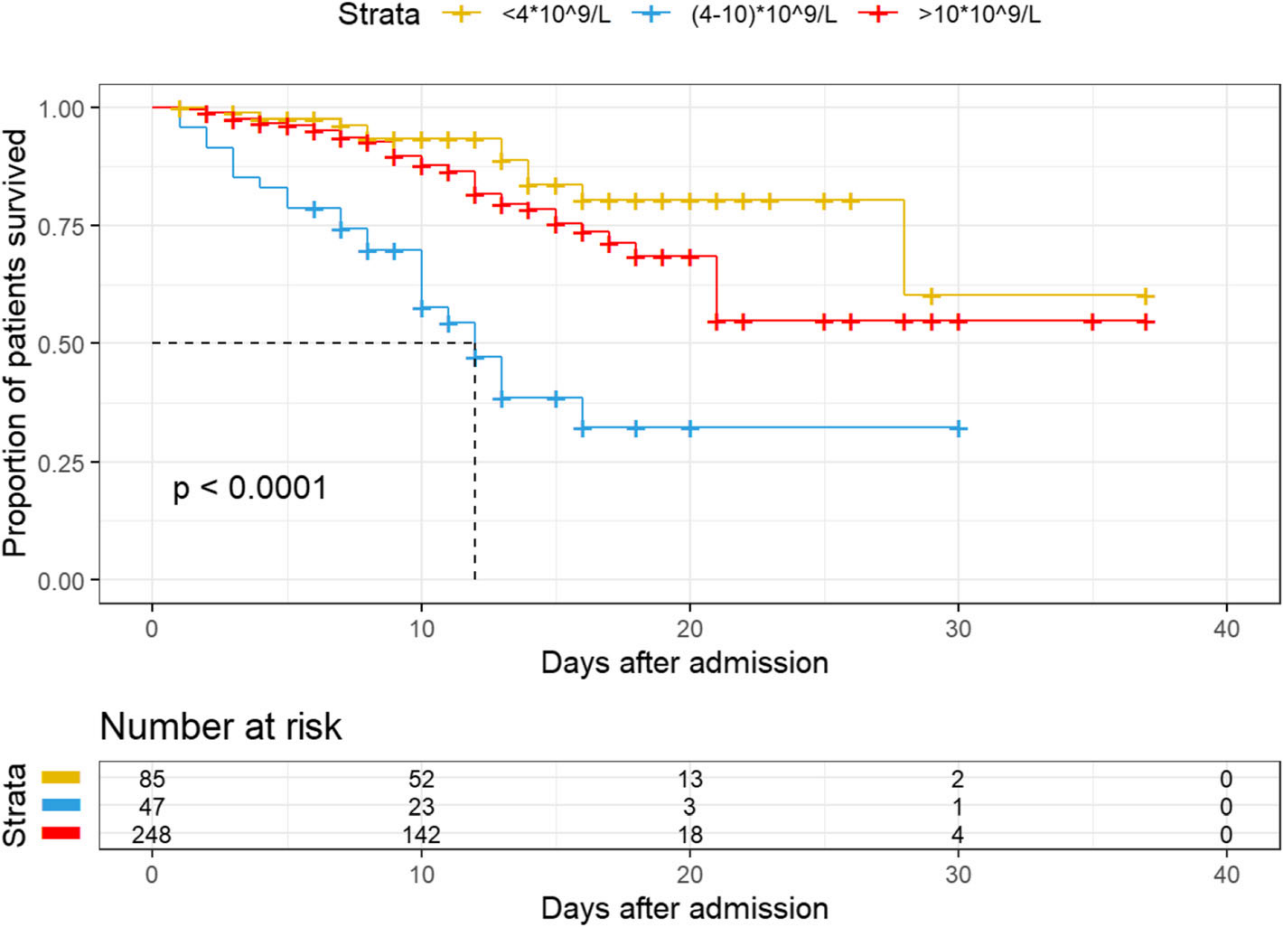
Kaplan-Meier Curves by White Blood Count Group

We included 380 patients with complete data for Cox regression. The univariate Cox proportional hazards model showed that the rate of death increased 1.072- fold with an increase in age (95% CI 1.055 to 1.089; *p* < 0.001). Patients were divided into two groups based on age with a cut-off point of 60 years. The Kaplan-Meier survival curves are shown in Fig. 1. In Fig. 1, it took 15 days for the probability of survival to drop below 50% for older patients while the probability of survival never fell below 50% for those age 60 or younger. The rate of death also increased with the presence of chronic diseases, such as diabetes [HR 2.187; 95% CI (1.361 to 3.515); *p* = 0.001], hypertension [HR 2.012; 95% CI (1.270 to 3.189); *p* = 0.003], and coronary heart disease [HR 4.195; 95% CI (2.198 to 8.008); *p* < 0.001]. The rate of death increased 1.187-fold in patients with an increased leukocyte count. The leukocyte count cut-off was at the upper limit of the normal value range. The Kaplan-Meier survival curves are shown in Fig. 2. In Fig. 2, it took 12 days for the probability of survival to drop below 50% for patients whose leukocyte count were between 4 and 10 ×10^9^/*L* while the probability of survival never fell below 50% for the other two groups. Other risk factors affecting death included lymphocyte count, and levels of creatinine, log transformed AST, log transformed glucose, as shown in Table 4.

**Table 4:** Analysis of factors affecting mortality using both the univariate and multivariable Cox proportional hazards model

	Univariate Model		Multivariable Model	
	Hazards ratio (95% CI)	*p* value	Hazards ratio (95% CI)	*p* value
Gender (male)	1.724 (1.103–2.694)	0.017	0.885 (0.533–1.470)	0.637
Age^#^	1.072 (1.055–1.089)	<0.001	1.066 (1.043–1.089)	< 0.001
> 60 years*	8.371 (4.701–14.910)	<0.001	-	-
Diabetes	2.187 (1.361–3.515)	0.001	1.148 (0.676–1.952)	0.609
Hypertension	2.012 (1.270–3.189)	0.003	0.852 (0.508–1.428)	0.543
Coronary heart disease	4.195 (2.198–8.008)	<0.001	1.081 (0.518–2.254)	0.836
White blood cell count ^#^	1.187 (1.138–1.238)	<0.001	1.135 (1.080–1.192)	< 0.001
>10 × 10^9^ per L ^&^	3.993 (2.469–6.459)	<0.001	-	-
Ln (Glucose)^#^	3.561 (2.221–5.708)	<0.001	1.844 (0.931–3.649)	0.079
Ln (AST)^#^	3.452 (2.448–4.867)	<0.001	1.480 (0.983–2.229)	0.060
High C-reactive protein(>10 mg/L)	18.960 (4.658–77.170)	<0.001	3.907 (0.900–16.970)	0.069
High Creatinine (>104 μmol/L)	3.430 (1.981–5.938)	<0.001	1.209 (0.637–2.294)	0.562
Low Lymphocytes (<1 × 10^9^ per L)	3.776 (2.150–6.635)	<0.001	1.516 (0.820–2.803)	0.185

*p* < 0 .05 was considered statistically significant

^#^ Per unit increase of the variable; * Reference group is patients age ≤ 60 years; ^&^ Reference group is patients white blood cell count ≤10 × 10^9^per L.

The multivariable Cox proportional hazards model was used to identify independent risk factors for mortality. We found that older age (HR 1.066, 95% CI 1.043 to 1.089) and higher white blood cell count (HR 1.135, 95% CI 1.080 to 1.192) were highly significant independent risk factors (*p* value < 0.01).

## Discussion

**Fig. 3:**
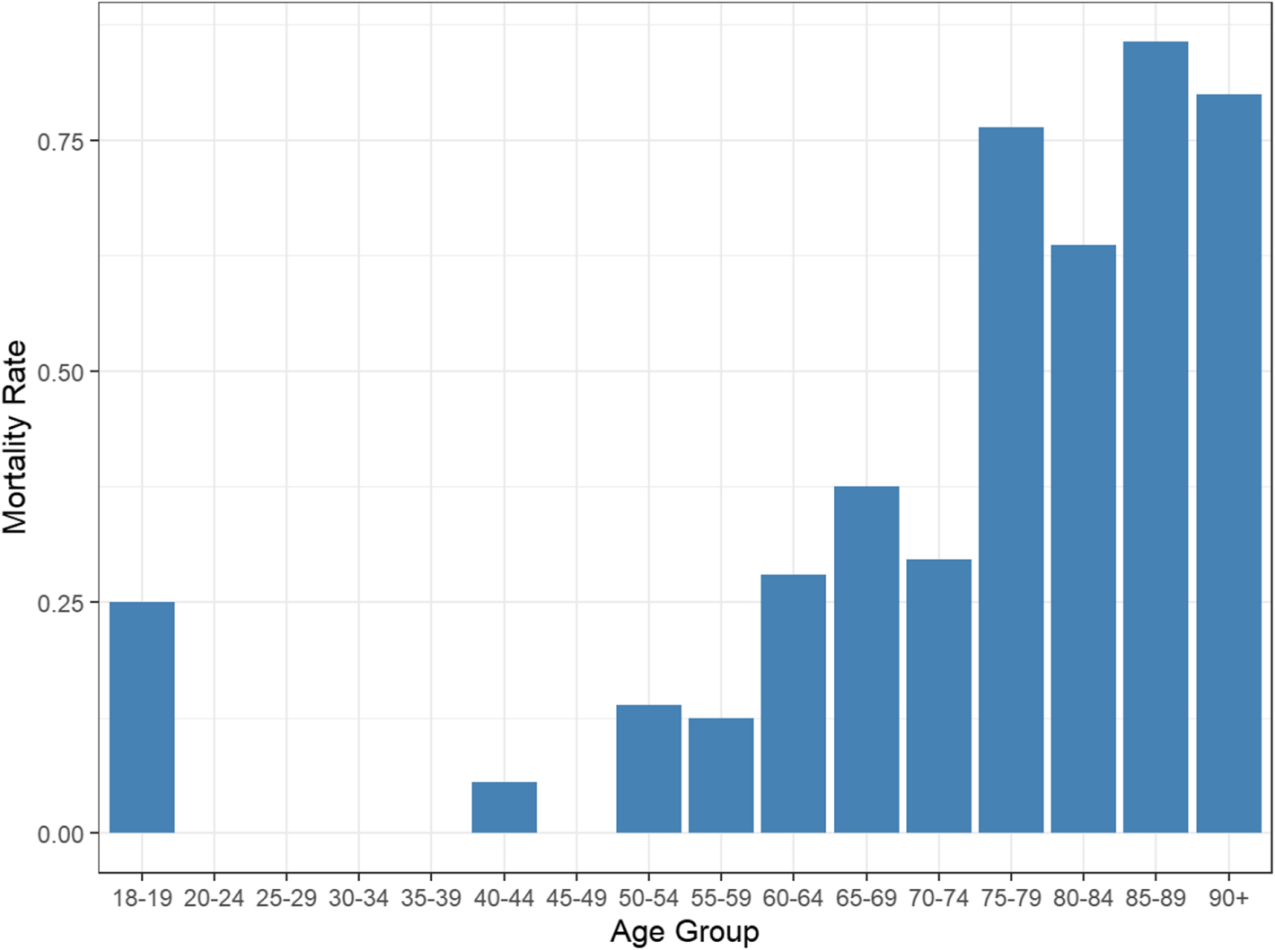
Mortality Trend over Different Age Groups

This retrospective study identified risk factors for death in severe and critical COVID-19 patients. We included 446 patients with COVID-19 in this study, and 20.2% of them showed a high mortality rate. As the disease is still in epidemic, our research cannot reflect the final mortality. In the study we found that both older age and higher white blood cell count were highly significant independent risk factors for death in hospitalized patients with COVID-19. In patients’ age more than 60 years, the death rate was significantly high. A study shows that humoral and cellular immune functions decline with age [14]. As the virus attacks the body, older people has an increased risk of death due to reduced immunity and the presence of co-morbidities. Zhou et al. [15] also reported that older age was an important independent risk factor for COVID-19 patients. We further divided all patients into different age groups (Fig. 3) and displayed the mortality trend over different age groups. From Fig. 3, we can observe an increasing trend of mortality as age increases. For patients who were at least 75 years old, the mortality rate is very high compared to other age groups. Note that there is only 1 out of 4 patients died in the age group 18–19. Due to small sample size, the interpretation should be in caution.

Results of laboratory tests demonstrated that higher white blood cell (WBC) count was a highly significant independent risk factor for mortality in severe and critical COVID-19 patients. We further divided the WBC into 10 deciles and calculate the Pearson correlation between mortality and the WBC count within each decile. By doing this, we expected to find out the association between mortality and WBC count. The results showed that the highest decile of WBC count has a correlation of 0.39 with mortality while the association in the other deciles were much lower. An elevated white blood cell counts often indicates an ongoing inflammatory responses to bacterial infection or disease progression. Patients with severe virus infection were more likely to co-infected with bacteria due to low immune functions. In other coronavirus infections such as SARS-CoV and MERS-CoV, could found “bcytokine storms” and immunopathology. Excessive inflammation could occur while immune response was dysregulated. The inflammatory response could stimulate the production of inflammatory cells and speed up the apoptosis of lymphocytes [16]. Patients with MERS found lower leukocyte and neutrophil counts, had signifcantly lower hazard ratios for mortality [17]. Recent study has reported that neutrophil-to-lymphocyte ratio was an independent risk factor for determining the in-hospital mortality in COVID-19 [18]. Therefore, we should pay attention to the indicators of inflammatory response at an early stage of the disease. White blood cell counts could be quickly obtained based on a routine blood routine test on admission, that may aid clinicians identify high-risk COVID-19 patients at an early stage. and administer combined antibacterial and antiviral therapy.

Some studies based on the characteristics of patients [15, 19, 20], showed a similar median age of onset of COVID-19. A few studies also revealed that men were more susceptible to COVID-19 [19–21]. Our study did not demonstrate significant gender differences, but in terms of patient mortality rate, males were at a higher risk of contracting COVID-19 than females, possibly due to a stronger innate and adaptive immune system providing resistance to viral infections in females [22–25].

Although the lymphocyte count of most patients was low [26–28], it was not found to be an independent risk factor in this study. The median lymphocyte count in patients who later died was significantly lower than that in surviving patients. Hence, lymphopenia might be related to viral invasion of the immune system leading to immune damage.

Prior to the first outbreak of SARS, a limited number of coronaviruses, causing only mild illnesses such as the common cold, were known to infect in humans [29]. Given the high prevalence, widespread distribution, extensive genetic diversity, and frequent genomic recombination of coronaviruses, it is likely that new coronaviruses will be regularly identified owing to frequent cross-species infections and occasional spillover events that occur in nature [30]. In the past two decades, major infectious diseases caused by coronaviruses included SARS, MERS, and the recent COVID-19. Therefore, it is necessary to follow up on the dynamics of this disease over time to have a better understanding of its pathogenesis and treatment strategies.

Our study has several limitations worth noting. Firstly, there was insufficient information on demographic characteristics, clinical symptoms, history of exposure, and personal history of the patients included in this study. Secondly, it was a retrospective study design and relied on data collected from case records. Information such as which patient comes from which hospital was not collected. There might be intracluster correlations among four hospitals, although we believe the impact on standard errors is minimal. Thirdly, the patients did not undergo all laboratory tests like neutrophil count, lactate dehydrogenase and serum ferritin tests. Therefore, their role might be underestimated in predicting death in hospitalized patients. Lastly, we studied a short-term prognosis of patients during hospitalization, however, SARS-CoV-2 is a newly discovered virus, and its onset, characteristics and prognosis are still being researched. So the validity of the risk factors for mortality derived from our cohort remains tentative, and we need a larger sample size and a longer follow-up period to validate our findings. In the later stages, follow-up of surviving patients must also be continued to better understand clinical course of the disease.

## Conclusions

The COVID-19 outbreak has been declared a global pandemic. In this study, we found that older age and higher white blood cell count were clinically useful independent risk factors for mortality of severe and critical COVID-19 patients in hospitalized. We hope this information will aid clinicians in the treatment of patients with COVID-19.

## Data Availability

The datasets used and/or analysed during the current study are available from the corresponding author on reasonable request.

## Abbreviations

COVID-19: Coronavirus disease 2019
SARS-CoV-2: Severe acute respiratory syndrome coronavirus 2
SARS-CoV: Severe acute respiratory syndrome coronavirus
MERS-CoV: Middle East respiratory syndrome coronavirus
SARS: Severe acute respiratory syndrome
MERS: Middle East respiratory syndrome
IRB: Institutional Review Board
ICU: Intensive care unit
CT: Computed tomography
RNA: Ribonucleic acid
IQR: Interquartile range
HR: Hazards ratio
CI: Confidence intervals
ALT: Glutalanine transferase
AST: Aspartate aminotransferase
BUN: Blood urea nitrogen
UA: Uric Acid
IL-6: Interleukin-6
CRP: C-reactive protein
HFNC: High-flow nasal cannula
NMV: Noninvasive mechanical ventilation
IMV: Invasive mechanical ventilation
WBC: White blood cell
